# Editorial: New Insights and Controversies in Diagnosis and Treatment of Adult Growth Hormone Deficiency

**DOI:** 10.3389/fendo.2021.819527

**Published:** 2022-01-10

**Authors:** Antonio Mancini, Maura Arosio, Ilonka Kreitschmann-Andermahr, Luca Persani

**Affiliations:** ^1^ Dipartimento di Medicina e Chirurgia Traslazionale, Università Cattolica del S.Cuore, Fondazione Policlinico Universitario A.Gemelli-IRCCS (Istituto di Ricovero e Cura a Carattere Scientifico), Rome, Italy; ^2^ Department of Clinical Sciences and Community Health, University of Milan, Milan, Italy; ^3^ Endocrinology Unit, Fondazione IRCCS (Istituto di Ricovero e Cura a Carattere Scientifico) Ca’ Granda Ospedale Maggiore Policlinico, Milan, Italy; ^4^ Department of Neurosurgery and Spine Surgery, University Hospital Essen, University of Duisburg-Essen, Essen, Germany; ^5^ Department of Medical Biotechnologies and Translational Medicine, University of Milan, Milan, Italy; ^6^ Division of Endocrine and Metabolic Diseases, IRCCS (Istituto di Ricovero e Cura a Carattere Scientifico) Istituto Auxologico Italiano, Milan, Italy

**Keywords:** growth hormone, hypopituarism, growth hormon therapy, growth hormone deficiency (GHD), growth hormone deficiency (childhood onset)

Growth hormone (GH) is a hormone whose biochemical actions go far beyond of what its name implies. Surely it is the main regulator of growth in childhood until puberty, however its metabolic functions are central even in adult age.

The history of medical use of GH now spans several decades. The growth-promoting action of extracts of anterior pituitary was already known when human GH was isolated in 1944. In the 1960s, further metabolic activities were discovered, strengthening the notion that it would be beneficial to replace the hormone in case of deficiency. GH, extracted from human cadavers at the time, was employed to improve longitudinal growth in hypopituitary children with severe growth retardation for nearly 30 years, despite the difficulties and costs associated with delivering this medication. When, in 1985, the association between cadaveric GH replacement and fatal, slow-viral Creutzfeldt-Jakob disease was uncovered, an important era in endocrinology seemed to have come to a sudden halt. Yet, recombinant technology, which made the synthesis of biosynthetic recombinant human GH (rhGH) possible since 1981 and was used first in GH-deficient children and then in adults, revolutionized this field and the life of these patients.

GH is produced by the pituitary gland in the somatotropic cells, which differentiate from mammosomatotroph lines, which explains the chemical analogy between GH and prolactin. It is the product of different GH genes present in chromosome 17: two GH genes, GH1 (or GH-N) and GH2 (or variant, GH-V), and three placental genes (also known as placental lactogens). GH-N is mostly expressed in the pituitary gland and, therefore, codes the circulating GH forms (22kDa and 20kDa), however it is also expressed in other tissues where GH can act as an autocrine and/or paracrine factor. Only about half of the GH is free in the circulation, the other half is tied up to a binding protein (GHBP). GHBP can be expressed in two different isoforms, of which the most abundant high-affinity one corresponds to the extracellular domain of GH liver receptor, and it is generated by tumor necrosis factor (TNFα)-converting enzyme (a metalloproteinase known as TACE), which represents another level of fine control of GH activity.

Its secretion is regulated in the hypothalamus in two ways: by means of a stimulatory hormone (growth hormone releasing hormone, GHRH) and an inhibitory one (somatostatin). Its actions are mediated by so-called somatomedins, produced in the liver and the periphery of the body. Its best known representative is the insulin-like growth factor-I (IGF-I). IGF-I is involved in a negative feedback loop with GH itself and is transported by a class of binding proteins (IGF-BP). Among these (six overall) the major binding protein in serum is IGF-BP3, that is controlled by GH and has been claimed as a possible marker of GH secretion, especially in the syndrome characterized by prolonged excessive GH secretion, acromegaly, and in pediatric GHD. IGF-I and IGF-BP3 are present in circulation in a 150 kDa ternary complex also including the acid-labile subunit (ALS) whose hepatic synthesis is also under the direct control of GH. The ternary complex significantly lengthens the half-life of IGF-I and constitutes its major deposit. IGF-BP5 is important for bone tissue formation and also forms ternary complex with IGF-I and ALS. On the contrary IGF-BP1 forms only small 50 kDa binary complexes and has been linked to metabolic status; insulin is its main regulator.

Twenty years ago, the classical view of the GH-IGF-I axis has been revaluated: the somatomedin hypothesis has been replaced by a more complex concept which takes into account GH action on different tissues, where it can induce local IGF-I synthesis. The major importance of the local autocrine/paracrine versus endocrine IGF-I actions on growth has been shown in models of mice knock-out for IGF-I genes in different tissues. All the pleiotropic effects of the axis are due to the coordinated action of GH (sometimes with a local production, as in bone, endothelium, heart or gonads) and IGF-I.

Furthermore, the model of a selective regulation of this axis at the hypothalamic level is a simplification and represents only a small part of a larger, more complex mechanism involving also peripheral organs. One of the most important modulator of GH secretion is ghrelin (also known as GH-releasing peptide or GHRP), produced by cells of the gastrointestinal tract, a powerful orexigenic peptide, physiologically released in the fasting state. Ghrelin action in turn is modulated by other factors, such as liver expressed antimicrobial peptide (LEAP)-2. Another newly discovered player is Klotho, a protein with antiaging properties in mice. In humans, Klotho is mainly expressed in the kidney, but also in the endothelium, where it induces nitric oxide (NO) production. Klotho is a regulator of GH secretion, as shown both in animals and humans, inhibiting negative IGF-I feedback on GH release; its levels are low in GH deficient subjects, however, the complex relationship with the GH axis is still to be clarified. The discover of ghrelin, LEAP-2 and Klotho, has thrown light on connections of peripheral organs and the pituitary.

Other regulatory factors are age-dependent, acting only in specific periods of life. One of them is growth differentiation factor (GDF)-15, directly produced by cardiomyocytes and related to growth disorders in children affected by cardiomyopathies; it inhibits the stimulation of IGF-I expression in the liver by GH, and therefore, contributes to the coordination of growth and cardiac function.

Normal aging is accompanied by a gradual decline in GH and IGF-1 levels. At 70, the secretion of GH in 24 hours is equal to 1/3 of that of a young adult (somatopause). The somatotropic axis influences the various epochs of life differently. While IGF-I excess could be dangerous for the mature man for the risk of favoring cardiovascular and oncological diseases, as observed in acromegaly, the rise of GH alone associated with reduced IGF-1/insulin activity observed in fasting and calorie restriction could be the key to explain the increased longevity of this condition.

The function of GH-IGF-I axis is strictly linked to nutritional status. Both GH secretion and effects are strongly regulated, directly or indirectly, by glucose, lipid and protein metabolism. Obesity is a condition of low GH secretory state, as demonstrated by blunted response to dynamic tests. Lower secretion in obese patients is the result of several factors, including the excess of circulating free fatty acids. Whatever the cause, in turn, it could contribute to the progressive metabolic alterations and co-morbidities, from dyslipidaemia to sarcopenia and to cardiovascular deterioration observed in obese patients.

The syndrome of adult GH deficiency (aGHD) has still poorly defined contours: it affects several organs and systems. It is currently considered uncommon, due to the non-specific and nuanced clinical presentation. Due to GH episodic secretion and short half-life, only provocative tests can play a diagnostic role. Organic causes (pituitary masses, iatrogenic after pituitary surgery or radiation therapy, trauma) are the most common. Idiopathic aGHD is a rare condition, even if its prevalence is probably underestimated, as highlighted in different cohorts in literature.

The example of obesity suggests that functional hyposecretion is a matter of fact and it is unknown if it can be assimilated to a real GHD at tissue levels. Circulating IGF-I concentrations are indeed normal in most obese subjects and GH sensitivity is increased probably due to hyperinsulinemia. Similarly, partial GH deficiency, with less clinical implications, yet significant, for instance at cardiovascular level, has been described.

Current guidelines on GHD diagnosis and treatment have been elaborated, however many questions are still debated. New tests for diagnosis have been recently proposed; moreover, non-conventional indications for diagnosis and treatment deserve further investigations. Controlled trials on beneficial effects on morbidity and mortality are still lacking and new formulations of GH are under investigation. Several questions are related to the age of affected patients (from transition age to ageing) and no clear indicators on how long therapy should be continued, are available. Other concerns are related to a possible pro-oncogenic effect, especially in patients who develop the deficiency because of a cancer or its treatment. The interrelations with other pituitary axes need further clarification since isolate GHD and multiple pituitary deficiencies may have a different clinical spectrum. Considering all these aspects, the aim of this Research Topic is to foster deeper insight in all questions related to aGHD, from molecular pathways involved in the pathophysiology to diagnostic tools and replacement therapy. Main advances in the papers, published in the Research Topic, are summarized and commented in this editorial.

## Growth Hormone Regulation

Devesa present a very interesting review on new insights about GH regulation, focused on three circulating hormones involved in the modulation of GH secretion, which are ghrelin, klotho and nesfatins, all basically acting at pituitary levels. Interestingly, the same hormones play a role in energy metabolism, and, in the case of klotho, also in mineral metabolism (which explains why the kidney is the main site of production). A detailed description of ghrelin synthesis, activation (in an acylated form), signalling and effects is presented. Interestingly, this activation is performed by an enzyme (GOAT) by attachment of a fatty acid, underlining the link of GH stimulation with nutritional fuels. Ghrelin colocalizes with GHRH neurons and therefore a hypothalamic mechanism is also likely. Circulating klotho is part of a transmembrane protein and is capable of stimulating GH release and antagonizing the inhibitory feedback on GH exerted by IGF-1. Some data are reported in humans, with reduced plasma levels of klotho in children and adults with GH deficiency, restored by replacement therapy. Finally, the role of nesfatins is far from being understood. Undoubtfully they exert a regulatory role in feeding behaviour, inhibiting food intake. Concerning GH physiology, they seem to be inhibitor of GH production by a downregulation of the cAMP/PKA/CREB signalling. They also can counteract the positive effect of ghrelin on GH synthesis and secretion.

## GH Deficiency: Aetiology and Clinical Management

Many questions arise about the transition period, which is very important from a metabolic point of view. Obviously, the role of GH in determining bone maturation is a main issue, but body composition and not secondarily sexual maturation are also affect by the hormone. Spaziani et al. review aspects, which are of fundamental importance in clinical management of childhood onset GHD. In the transition period, GH replacement appears critical for the achievement of an adequate peak bone mass; however, equally clear is the primary role of rhGH on body composition and metabolic profile and, probably, in the achievement of a complete gonadal and sexual maturation. The most relevant issue in the transition period is the high rate of spontaneous recovery of GH function after the achievement of final height. A percentage between 25% and 100% of subjects with previous GHD diagnosis during childhood display an adequate response when undergoing re-testing, thus raising several questions on a) the opportunity to treat children with partial GHD, b) when performing the re-testing; c) which is the most adequate test and relative cutoff for the confirmation of GHD. In Table 1 of their orginal publication, Spaziani et al. provide the list of the available GH provocative tests and related cutoffs.

Traumatic brain injury (TBI) ranges among the those aetiologies of hypopituitarism and, thus, aGHD, which are inbetween recognized as classical causes, as reviewed by Gasco et al. in his overview on post-traumatic hypopituitarism. First described in 1918, it was, for a long time, considered a rare sequel of trauma. However, the situation has radically changed in the last two decade and the prevalence of TBI is, nowadays, estimated between 27.5 and 32%. GH, together with gonadotropins, seems to be the most frequent hormone involved. The physiopatologic mechanisms underlying this condition are comprehensively described, according to their division into two distinct periods of time: the primary brain injury at the time of trauma, with direct damage of neural structures and hypoadrenalism as the main life-threatening hormonal deficit; and the secondary one, which is based on different mechanisms, such as excitotoxicity (mainly related to glutamate), secondary ischemia (considering the peculiarity of pituitary vascularization) and inflammatory response; the latter also including autoimmune mechanisms and, possibly, a genetic vulnerability. The diagnosis is based on the same tests used in other aetiologies of GHD, but with some peculiarities (for instance the risk of seizures after ITT) and the lack of hypothalamic derangement sensitivity when using GHRH plus arginine. Glucagon in this case seems to be the gold standard and 6-12 months after trauma appears to be the ideal period to perform GH test. The rationale for beneficial effects are based on pleiotropic actions of GH-IGF-1 axis on neurogenesis and neurorepair. Anabolic GH functions could also be a key point for the recovery. However, clinical studies are still heterogeneous, so that conclusive data are still far to be obtained. The authors underline that postTBI GHD, compared with GHD secondary to non-functioning pituitary adenomas, seems to exhibit a less severe biochemical picture, but worse quality of life (QoL) scores. The QoL improvement after GH replacement therapy seems to have a principal effect and was shown to be maintained for a long period, up to eight years.

The group of Giavoli provides and overview on the management of GHD patients with multiple pituitary deficiencies (MPHD). A condition of untreated GHD masking concomitant pituitary deficiencies, mainly central hypothyroidism and hypoadrenalism, is now a consolidated concept. Therefore, thyroid and adrenal functions should be soon re-tested after the introduction of rhGH replacement. In their manuscript, Profka et al. give information on the possible contexts in which GHD may develop and examining the proposed mechanisms at the basis of interactions between the GH/IGF-I system and other axes. A relevant part of the manuscript is dedicated to the sexual dimorphism of GH-IGF1 function and on the possible role of rhGH in the induction of fertility in different clinical conditions in both sexes.

## Effects of GH Replacement Therapy

### GHD and Cardiovascular Risk

The issue of GHD, insulin resistance and cardiovascular risk is the topic of two articles in this Research Topic. In the first, van Bunderen et al. present data from a clinical trial in which they investigated the effects of GH dose titration to low-normal or high-normal levels of IGF-I on (micro)vascular function, insulin resistance and body composition in order to explore the mechanisms underlying the U-shaped relation of IGF-I levels with cardiovascular disease. Based on the knowledge, that epidemiological data give evidence for a bidirectional link between serum IGF-I concentrations and cardiovascular disease with an increased cardiovascular risk (CVR) in states of aGHD but also in acromegaly, they investigated 30 patients with aGHD on GH replacement, titrated to low vs. high-normal IGF-I levels. They found that an increase of GH dose with subsequent high-normal IGF-I levels led to a reduction in waist circumference, but also to a significant increase in insulin resistance. Also, neurogenic and endothelial vasomotion domains were affected by a change in GH dose, paralleling, in part, the changes in waist circumference. They concluded from their results that higher IGF-I levels may be beneficial for body composition but seem to be detrimental in terms of insulin resistance. While van Bunderens results must be considered preliminary and do not allow, at present, to provide clear dosing strategies, they open up avenues for further research in this important field.

In the second article, Ren et al. introduce a further potential player into the intricate relationship between GHD, CVR and insulin resistance. Based on the knowledge that patients with aGHD have elevated levels of circulating inflammatory factors, accompanied by increased levels of oxidative stress and endothelial dysfunction, they explored levels of mesencephalic astrocyte-derived neurotrophic factor (MANF) in aGDH patients and normal controls. MANF is a secreted stress-response protein with selective protective effects on dopamine neurons and immune modulatory properties, which serves as a regulator of metabolic homeostasis. 101 aGHD patients and 100 matched healthy controls were included in the analysis. The authors found that circulating MANF content of aGHD patients was significantly lower than in the controls and that, moreover, MANF levels were linearly correlated with homeostasis model assessement)-insulin resistance (HOMA-IR) in the aGHD population. Those patients with MANF at the lowest concentration tertile, had a significantly higher disease odds ratio, Framingham risk socre and 10-year-risk of atherosclerotic cardiovascular disease than the hightest concentration tertile. In sum, the authors were able to show that MANF is strongly associated with insulin resistance and abnormal lipid metabolism under aGHD conditions. Thus, in the future, MANF may play a role in aGHD diagnosis and even provide therapeutic potential for later cardiovascular disease.

GHD in elderly is a particular topic which is addressed by two papers in the Research Topic. Ricci Bitti et al. performed a minireview about the peculiarity of clinical presentation, diagnosis and outcomes of aGHD patients in this period of life. Due to the similarity of the ageing process and GHD symptoms, the diagnosis of aGHD in older patients is particularly complex, since no clear adjustments for diagnostic cut-offs in GH dynamic test are available. There is agreement to start therapy at low doses and up-titrate according to clinical response, including IGF-I levels, which should be maintained between -1/-2 and +1/+2 DS for age, monitoring of metabolic parameters and of side effects, which could be more harmful in elderly people. Few randomized and controlled studies have been reported, which are still inconclusive due to the number and heterogeneity of patients; moreover, no data are available about efficacy and long-term therapy in patients above 80 years. Greater attention should be placed on cardiovascular morbidity and mortality and on cognitive function. Despite no clear evidence is reported on increase of muscle strength, there is sufficient suggestion that GH may reduce its age-related decline. However, in the authors’ opinion, the main goal of GH replacement in the elderly should be the improvement of QoL, in turn related to frailty and the risk of loss of independence, typical of the ageing; they underline the importance of personalized treatment and careful follow-up.

The other is a single-centre observational study (Scarano et al.) which gives an important experience, selecting a group of GHD patients treated for 7 years, comparing the effects of therapy in groups divided according to age (10 elderly and 29 adult-onset GHD); they were recruited by a large cohort of 196 hypopituitary patients, with an inclusion criterion of this therapy period; a comparison with age-matched control group is also presented. According to concepts above described, the mean GH dose was lower in the elderly group, but with the same aim to maintain IGF-levels in the normal range for the specific age. The study shows that the effects on body composition are more evident in AGHD (reduction of waist and hip circumferences and waist-hip ratio) than in EGHD (that showed only reduction in hip circumference): similarly lipid profile was improved more in AGHD (decreased in total and LDL-cholesterol and triglycerides and increase in HDL-cholesterol) than in EGHD (only triglycerides significantly decreased). An increase in morning glycemia was observed only in AGHD, but without modification of HbA1c. EGHD showed, as expected, higher systolic blood pressure, which however did not significantly change after treatment. Interestingly the prevalence of diabetes mellitus did not differ from that of general population; a risk of develop it could be related to impaired glucose homeostasis in obese GH adults. On the contrary, the authors showed a higher prevalence of dyslipidaemia in adult controls than AGHD. The prevalence of Metabolic Syndrome is increased in AGHD during treatment, due to the increase in glucose levels, BMI and systolic blood pressure in this long-term study. In agreement with other study, the main conclusion was the beneficial effect on body composition and lipidic pattern, less pronounced, but present, also in elderly GHD people.

In this context, Chen et al. performed a meta-analysis to evaluate the efficacy and safety of weekly long-acting growth hormone (LAGH) replacement therapy, a new frontier for GHD, compared to daily growth hormone in children with short stature. This analysis reveals that LAGH has no significant difference compared to daily growth hormone in children with short stature on several clinical parameters (height velocity, final height SDS, bone age, IGF1-SDS, as well as on incidence of adverse events). This is of significance in medical practice due to the various nuisances of daily injection in adherence to treatment, for example, as the authors well defined in the introduction. One possible counfounding factor in this analysis is the availability of six different LAGH formulations, each one tested in a small number of patients so far. Despite the limited number of children treated with LAGH, the meta-analysis would indicate that both short- and long-acting rhGH formulations can be used without major consequences on children’s growth or on their side effects.

## New Therapeutic Issues

The challenges about GH treatment in adults concern the entire lifespan (from transition to aging). The metabolic role indicates that it is not simply a growth hormone; nevertheless it is not a antiaging therapy. A precise definition of GH deficiency in different clinical situations is mandatory before starting treatment. Another open question which could be of interest in clinical management regards the objective evaluation of patients’ adherence.

Few studies have investigated the adherence to GH therapy in the adult GHD population and the psychological reasons that influence it. In children a review showed that up to 71% of the young patients were non adherent to their GH medication. The group of CJ Strasburger and I Kreitschmann-Andermahr, on behalf of the German PATRO Board, studied this important and overlooked aspect in depth using for the first time in GHD a methodology already well validated in other chronic diseases. Using specific questionnaires they analyzed three major psychological domains, that is: strategy of coping with their chronic disease, beliefs about medications and quality of life and related them to adherence to GH therapy. Their series consists of 107 patients (53% M, mean age 50 years) with severe GHD in almost all cases from organic causes, followed up in 5 German referral centers and in stable current therapy with rhGH. The AA note that the majority of the patients had high rhGH specific adherence scores and are strongly convicted of their need for GH medication. In addition the AA find that active coping is the most common adaptation strategy, and the one that most correlates with adherence to therapy; that most patients judge the benefits of rhGH greater than the potential negative effects, with only 4 patients whose fear for side effects outweighs the perceived benefits.

Of particular interest is the evaluation of QoL in these patients: in fact, if there are many studies that have shown severe QoL impairment in untreated GH-deficient patients in respect to the general population, very few have evaluated it in the course of replacement GH therapy. Well, the physical QoL remains reduced by more than 1 SD in 13% and by more than 2 SD in approximately 7% of these patients, and, surprisingly, mental QoL in 12% and 25%, respectively. This shows a severe mental impairment, not related to age, in a large proportion of the investigated patients, which is mainly due to a reduced vitality and a bad perception of the one’s general health status. Noteworthy, the adherence to therapy is negatively correlated to the mental QoL, conversely, a lower physical QoL, as observed in the oldest patients, correlates with higher adherence to therapy.

Although the study did not include a control group of untreated patients, as the questionnaire used (SF-36) is the same, some comparisons can be made with historical series of untreated GHD patients showing overall better QoL of patients with hypopituitarism on replacement GH therapy. The important observation remains that those patients with impaired mental QoL often demonstrate a depressive coping and also have a lower adherence to therapy, as if they are less able to translate their belief in the usefulness of therapy with GH into action and these will be the patients clinicians need to recognize and to focus their efforts on in the future.

Yuen et al. provide an excellent overview on the present situation of long-acting GH (LAGH) analogs, the development of which has been prompted by issues of patients’ non-adherence to the presently approved daily recombinant human GH (rhGH) preparations. LAGH analogs that allow for a decreased injection frequency may offer increased patient acceptance, tolerability and therapeutic flexibility. However, the authors also point out that there may be pitfalls associated with these LAGH analogs, among them an unphysiological GH profile and different molecular structures that might pose clinical problems in terms of dose initiation, therapeutic monitoring, incidence and duration of side-effects and long-term safety. Moreover, the technology used to prolong GH action may cause fluctuations of peak and trough serum GH and IGF-I levels and variations in therapeutic efficacy. Non-inferiority to daily rhGH has already been proven for some LAGH analogs, not only in terms of increased growth velocity but also improved body composition in children and adults. With two LAGH analogs marketed in Asia, one recently approved in the United States, one more approved but not marketed in Europe along with several others proceding through various stages of clinical development, there seem to be exciting new treatment opportunities for pediatric and adult GH indications at the horizon. However, the authors caution that long-term surveillance of safety and efficacy of LAGH analogs are needed to establish their worth in clinical practice.

## Concluding Remarks

GHD syndrome has still poor defined features and many unsolved question. Due to new discovered function of GH, the search for clinical/biochemical parameters, which could be useful in risk prediction, is yet to be expanded.

The possible role of GH in other diseases, such as osteoporosis, infertility, cardiac failure and many more, could represent a “non-conventional” indication to perform dynamic GH tests unveiling masked and underestimated GHD. Therefore, it has also the aim to sensitize physicians, who are not familiar with this Research Topic, to extend their cultural interest and clinical practice in GH physiopathology.

## Author Contributions

AM, MA, IK-A, and LP wrote sections of the manuscript. All authors contributed to manuscript revision, read, and approved the submitted version.

## Funding

Partially supported by the Ricerca Corrente Funds of Istituto Auxologico Italiano (IPOFICTUS; code: 05C303_2013).

## Conflict of Interest

The authors declare that the research was conducted in the absence of any commercial or financial relationships that could be construed as a potential conflict of interest.

## Publisher’s Note

All claims expressed in this article are solely those of the authors and do not necessarily represent those of their affiliated organizations, or those of the publisher, the editors and the reviewers. Any product that may be evaluated in this article, or claim that may be made by its manufacturer, is not guaranteed or endorsed by the publisher.

